# The role of breed and personality descriptions in influencing perceptions of shelter dog adoptability

**DOI:** 10.1017/awf.2025.10043

**Published:** 2025-11-17

**Authors:** Courtney Archer, Nathaniel J Hall, Allison Andrukonis

**Affiliations:** 1 University of Minnesota, Department of Animal Sciences, 1364 Eckles Ave, St Paul, MN, 55108, USA; 2 Texas Tech University, Department of Animal and Food Sciences, 1308 Indiana Avenue, Lubbock TX 79409, USA; 3 University of Wisconsin-Madison, Department of Animal and Dairy Sciences, 1675 Observatory Dr, Madison, WI, 53706 USA

**Keywords:** Adopter preferences, animal welfare, behavioural descriptors, breed identification, canine adoption, municipal shelter, public perception

## Abstract

The majority of dogs in US animal shelters are of mixed breed. Many animal shelters still use visual identification to assign breed labels, despite research indicating it to be largely inaccurate. Some shelters now include personality descriptions in conjunction with, or instead of, breed labels. However, little is known about the interaction between these factors. Thus, the aim of this study was to experimentally evaluate the impact of breed labels and descriptions on the perceived adoptability of dogs. Participants, recruited both in-person at a shelter and online, were shown ten dog photos, and indicated how likely they were to adopt the dog. The photos were randomly presented under four conditions: (1) photo only; (2) photo with breed label; (3) photo with description; and (4) photo with both a breed label and description. Overall, descriptions significantly increased perceived adoptability, while breed labels decreased it. Certain breed labels, such as ‘Chihuahua mix’, ‘Chow mix’, ‘Jack Russell Terrier mix’, ‘Miniature Pinscher mix’, and ‘Terrier mix’, negatively impacted adoption ratings, while ‘Lab mix’ had a positive effect. Descriptions like *affectionate*, *calm*, *eager to make you proud*, *easy-going*, *friendly*, *lively*, *non-dominant*, and *sociable* improved perceived adoptability, whereas *energetic* reduced adoptability. There were no significant interactions between breed labels and descriptions. Additionally, there was substantial individual participant variability in adoption interest across photos. These findings suggest animal shelters might increase adoption interest in dogs by removing breed labels and including positive descriptions in dog adoption profiles. Such changes may contribute to improved animal welfare by reducing shelter length of stay.

## Introduction

Approximately 3.1 million dogs enter around 7,000 animal shelters across the US annually (American Society for the Prevention of Cruelty to Animals [ASPCA] 2019; Woodruff & Smith 2019). The majority of these dogs are not of a singular breed (Patronek *et al.* 1995; Posage *et al.* 1998; Gunter *et al.* 2018). In a study of 919 shelter dogs across two different US shelters, Gunter and colleagues (2018) found that only 4.9% of the dogs were genetically identified as belonging to a single breed. Despite the infrequency of dogs of a singular breed, animal shelters typically assign dogs a breed label to aid in internal organisation and public communication. Although DNA testing for breed identification exists, it is cost prohibitive. Thus, animal shelter staff primarily use visual identification to determine breed labels (Hoffman *et al.* 2014; Olson *et al.* 2015).

Visual identification typically involves assessing phenotypic traits such as coat colour and length, height, musculature, and weight (Hoffman *et al.* 2014; Gunter *et al.* 2018). However, previous studies suggest this method to be unreliable, even among dog professionals (Olson *et al.* 2015). For example, in a study of 384 shelter dogs, staff correctly identified at least one breed in 67.7% of cases, but accurately identified both the primary and secondary breeds in only 10.4% of dogs (Gunter *et al.* 2018). It is also worth noting that 55.6% of the primary and secondary breed matches were purebred dogs. In another study on visual identification in shelter staff, Voith and colleagues (2009) revealed that only 25% of visual breed identifications by adoption agencies matched the predominant breeds identified by DNA analysis, with 87.5% of the dogs not having all the visually identified breeds detected genetically. Additionally, a study of 923 participants from various dog-related professions found that fewer than 50% were able to correctly identify even one breed present in fourteen of the twenty dogs tested based on genetic results, and fewer than 1% correctly identified a breed in six of the dogs (Voith *et al.* 2013). They also found a very low inter-observer reliability (α = 0.23) amongst participants, suggesting that not only were participants poor at identifying breeds, but they were also inconsistent.

Not only are breed labels frequently inaccurate, but breed labels can impact the dog’s welfare through decreasing their perceived attractiveness and adoptability and increasing their length of stay in the shelter (Gunter *et al.* 2018; Weseley-Jones 2022). More specifically, dogs labelled as certain breeds such as ‘pit bulls’ or pit bull types may have a significantly longer length of stay in the shelter or even be euthanased (Lepper *et al.* 2002; Protopopova *et al.* 2012; Gunter *et al.* 2016; Patronek & Crowe 2018). Given the high proportion of mixed breed dogs, inaccuracy of breed labelling, and negative connotations of certain breeds, some animal shelters have stopped breed labelling. Cohen and colleagues (2020) found that eliminating breed labels from adoption cards in three New York animal shelters significantly reduced the median length of stay for dogs from 30.3 to 19 days. Additionally, the shelters did not see an increase in dogs being returned after breed labels were removed (Cohen *et al.* 2020).

In order to provide potential adopters with more information about the dogs, animal shelters have started adding personality descriptions instead of, or in conjunction with, breed labels (Nakamura *et al.* 2019; Markowitz 2020). These descriptions may offer potential adopters more information about the dogs as well as help shelter staff promote the dogs more effectively. Additionally, with an increase in the online promotion of adoptable animals, descriptions might play a crucial role in attracting potential adopters (Weiss *et al.* 2012; Workman & Hoffman 2015; Markowitz 2020; Kelling *et al.* 2024). In an exploration of over 70,000 online dog adoption profiles, Nakamura and colleagues (2019) found that description terms like *independent*, *lively*, *eager*, and *clever* were associated with shorter lengths of stay, while terms like *only dog*, *dominant*, and *sensitive* were linked to longer lengths of stay. This suggests that the specific words used in the description might have a significant impact on the likelihood of adoption.

A lot of the research into the impact of both breed labelling and descriptions on dog adoption has been correlational. However, a recent study aimed to experimentally evaluate not only how breed labelling impacted perceived attractiveness, but also how a descriptor, *affectionate*, might interact with the breed label (Weseley-Jones 2022). Participants viewed a photo of a dog labelled as either ‘Pit Bull’, ‘Mixed Breed’, ‘Affectionate Pit Bull’, or ‘Affectionate Mixed Breed’ and answered a five-item questionnaire to attain an overall attractiveness score. Participants viewed the image of the dog labelled ‘Mixed Breed’ as significantly more attractive than the dog labelled ‘Pit Bull’. However, there was no interaction between breed label and the descriptor. It is worth noting that the study only utilised one photo and one descriptor and did not have a condition without any breed label or description, which limits the generalisability of the findings.

Thus, the present study aimed to address gaps in existing literature by experimentally evaluating the combined and independent effects of breed labels and descriptions on perceived adoptability of shelter dogs. We hypothesised that: (1) the same dog will be perceived as significantly more adoptable when presented without a breed label compared to when a breed label is included; (2) dogs with specific breed labels (e.g. pit bull mix) will be perceived as significantly less adoptable compared to other breed labels (e.g. lab mix); and (3) dogs with a description will be perceived as significantly more adoptable.

## Materials and methods

### Participants and procedures

All procedures were reviewed and approved by the Texas Tech University Institutional Review Board (IRB2019-777). Participants were categorised into two populations: in-person and online. The in-person participants were individuals who visited a municipal shelter in the South-Central US. In-person participants (n = 103) were recruited from March 5^th^, 2020, to March 14^th^, 2020. The online participants (n = 1,113) were recruited through widespread online promotion from March 15^th^, 2020, to March 30^th^, 2020. Online promotion occurred through personal and professional social media sites, email, word of mouth, and flyer advertisement. Each participant gave informed consent prior to beginning the survey. Participants were informed that their responses would remain anonymous, that the data would be used for research purposes only, and that they could withdraw from the study at any time without penalty. The survey consisted of 19 items: nine demographic questions and ten experimental prompts and took approximately five minutes to complete. The demographic section included questions about age, gender, relationship status, children, and dog ownership. The ten experimental prompts, which are explained in detail below, examined the potential influence of breed labels and descriptions on adoptability ratings of dogs.

### Survey design

**Figure 1.**
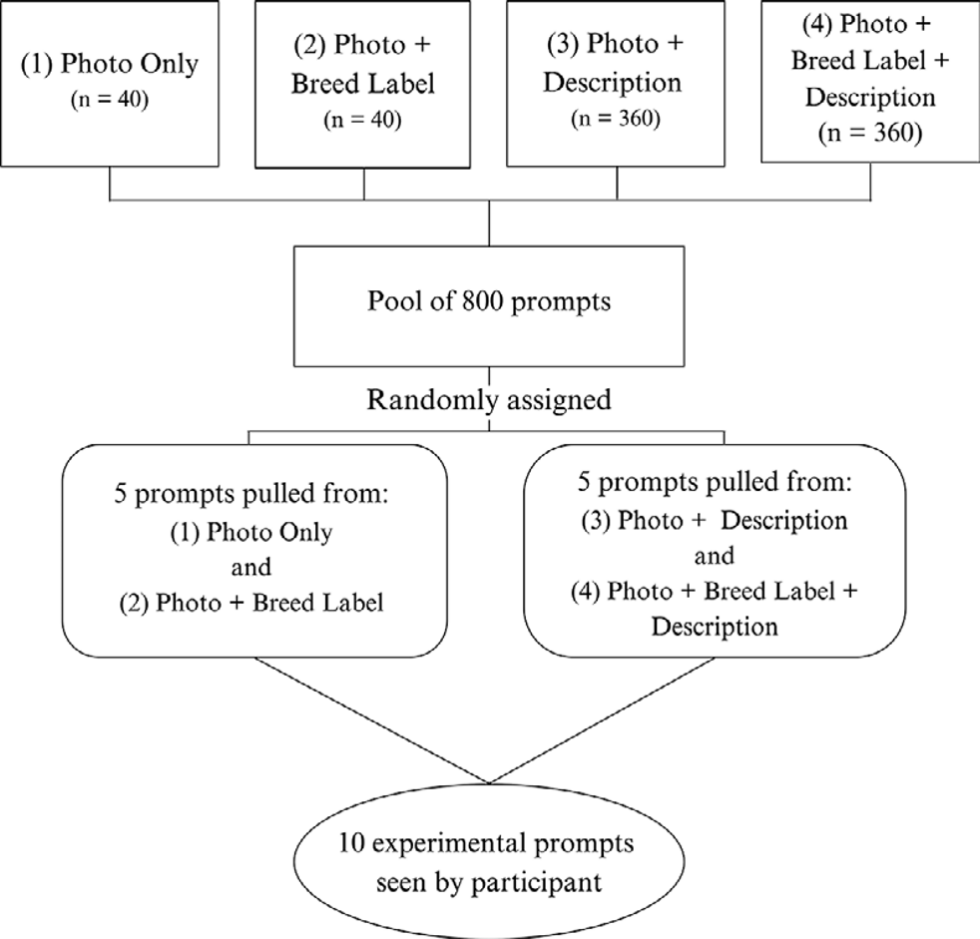
A breakdown of the prompt matrix. Each participant saw ten randomly assigned experimental prompts. The prompts were pulled from a pool of 800 potential prompts.

A pool of 800 prompts was created through combining 40 photos, 20 breed labels, and nine personality descriptions in Qualtrics^XM^ Online Survey Software. Of the 800 prompts, each participant viewed a survey comprised of ten randomly distributed experimental prompts derived from four distinct categories: (1) Photo Only; (2) Photo + Breed Label; (3) Photo + Description; and (4) Photo + Breed Label + Description. Of the ten prompts, participants received five randomly pulled from (1) Photo Only and (2) Photo + Breed Label. The remaining five prompts were from (3) Photo + Description and (4) Photo + Breed Label + Description. See Figure 1 for a visual representation of the prompt distribution. Although a participant could see a specific breed label or description twice during their survey, they could only see a specific photo once. In all prompts, participants answered the same question: *How likely are you to adopt this animal?* The participants answered the question on a sliding scale with parameters of 0–100. Numerical anchors were added on the scale to inform the participants how to rate the animal. Descriptor anchors were also provided at 0, 50, and 100. These anchors read, *No Thanks*, *Maybe*, and *Yes Please*, respectively. All questions included a photo of a dog, with the ‘photo only’ condition serving as the control against which the effects of breed labels and descriptions were compared.

The photos used in this survey were private pictures taken at a local municipal shelter of dogs that were up for adoption at the time. Based on the literature on photo characteristics and adoption, each photo was high quality, a straighton head shot with the dog looking at the camera, taken outside, and with no unique features (Lampe & Witte 2015). Pictures can be seen in the Supplementary material. Similar to how animal shelters typically determine breed labels, the breed labels in the current study were determined by three members of the research team based on the dog’s physical appearance. Each breed label included the assumed dominant breed of the dog followed by the word ‘mix’. In order to mimic how breed labels are assigned in shelters, breed labels were based solely on visual assessment by the research team; no genetic testing was conducted to confirm breed composition. All dogs presented in this study were believed to be mixed breed dogs. The breed labels and their associated photo can be seen in the Supplementary material. Each photo was consistently paired with the same breed label across all survey conditions to maintain internal consistency and avoid implausible or incongruent pairings.

### Descriptions

In this study, the descriptions resemble marketing-style behavioural descriptions commonly used in adoption profiles. These descriptions were not the result of formal behavioural testing. The nine descriptions included in the present study were informed in part by Nakamura and colleagues (2019). The descriptions were randomised so a dog could have a different description in different surveys. Each of the 20 breeds of dogs were labelled with all nine descriptions in the survey pool. An example of each description is provided below.

1. *Affectionate*: This dog is Affectionate. He loves to give kisses and lay in your lap. This dog will follow you around everywhere.
2. *Calm:* This dog is Calm. He loves to cuddle and sleep with you. This dog patiently waits for you to return home so he can get all the belly rubs.
3. *Eager to Make You Proud*: This dog is Eager to Make You Proud. He can learn any trick in the book with just a little motivation with treats. This dog loves to make people smile.
4. *Easy-Going:* This dog is Easy-Going. He is up for anything, long naps on the couch, slow walks around the neighbourhood, or even car rides around town. This dog is happy as long as he gets to be around you.
5. *Energetic*: This dog is Energetic. He loves to go on long runs, hikes, and adventurous journeys. This dog would love a big backyard and many toys to play with.
6. *Friendly:* This dog is Friendly. He loves to give kisses and play with everyone he meets. This dog is well behaved around others and loves to play with small children.
7. *Lively:* This dog is Lively. He enjoys long walks, meeting new people, and saying hello to every animal or human that walks by. This dog will make an adventure out of any day.
8. *Non-Dominant*: This dog is Non-Dominant. He is easily trained and loves to follow directions. This dog loves all other dogs
9. *Sociable*: This dog is Sociable. He loves to meet everyone and anyone who comes near to give a friendly hello. This dog loves to play at the dog park and socialise with other pups.

### Experimental unit prompts

1. Photo Only

The 40 possible Photo Only prompts only provided a picture of a dog. Each potential prompt had a different dog. No text was provided in the Photo Only prompts.

1. Photo + Breed Label

The 40 possible Photo + Breed Label prompts provided a picture of the dog as well as a breed label associated with it. The breed labels could be used more than once, but the picture of the dog always had the same breed label associated with it.

1. Photo + Description

The 360 possible Photo + Description prompts provided a picture of a dog with one of the nine descriptions discussed above. Each description was combined with all 40 pictures.

1. Photo + Breed Label + Description

The 360 possible Photo + Breed Label + Description prompts provided a picture of a dog, the breed label associated with that dog, and one of the nine descriptions listed above. Each photo and description were used only once per participant. This approach minimised the potential for bias due to repeated exposure and ensured that each evaluation was based on unique and independent stimuli.

### Data analysis

All data cleaning and analyses were run in R (R Core Team 2024). Prior to data analysis, participants (n = 45) who did not answer at least one experimental question were removed from analysis, leaving 1,171 participants in the final analysis. To evaluate the effect of breed label and personality description on the probability of adoption, we fitted a linear mixed effect model (Kuznetsova *et al.* 2020). Adoption likelihood rating was predicted by presence/absence of the breed label, type of description (e.g. *energetic*, *affectionate*, none, etc), their interaction (breed label × description), and the location of the survey (online or in-person in the shelter). Note that although the descriptions could be randomised across photos, breed labels could not. Thus, breed labelling was coded simply as present/absent, because breed is inherently associated with a picture in this study due to the morphological limitations of providing an image and breed label (e.g. an 80 lb dog could not be labeled as a ‘Chihuahua Mix’). A random intercept was fitted for the participant ID and for the picture ID.

To explore whether a breed label had a specific effect on certain breed mixes, we conducted independent regression in which the adoptability rating was predicted by the presence/absence of a breed label for each breed separately (Lenth *et al.* 2024). Due to the exploratory nature of the study, we retained a less conservative criterion of statistical significance of *P* < 0.05 (i.e. we did not lower our statistical criterion for multiple testing).

Lastly, we explored the effect of individual photos. The mean adoptability rating of each photo was calculated as well as the bootstrap estimated 95% confidence intervals. This was then used to explore whether individual photos were generally rated as more ‘adoptable’ than others.

## Results

The majority of participants identified as female (88.1%), were between the ages of 18–25 (32.5%), married (46.7%), did not have children (58.8%), and owned at least one dog (83.8%). Additionally, most participants took the survey online (91.3%). Table 1 shows a full breakdown of participant demographics. The mean adoptability ratings for breed labels and descriptions are presented in Table 2.

**Table 1.** Demographic characteristics of survey participants (n = 1,171)

Characteristic	n (%)
**Gender**	
Female	1,032 (88.1)
Male	134 (11.4)
Genderqueer or nonbinary	4 (0.3)
Prefer not to say	1 (0.1)
**Age**	
18–25	380 (32.5)
26–35	279 (23.8)
36–45	188 (16.1)
46–55	153 (13.1)
> 55	168 (14.3)
Prefer not to say	3 (0.3)
**Marital status**	
Single	349 (29.8)
Dating	199 (17.0)
Married	547 (46.7)
Divorced	68 (5.8)
Prefer not to say	8 (0.7)
**Children**	
Yes	475 (40.6)
No	689 (58.8)
Prefer not to say	7 (0.6)
**Current dog ownership**	
0	189 (16.1)
1	359 (30.7)
2	317 (27.1)
3	176 (15.0)
> 4	129 (11.0)
Prefer not to say	1 (0.1)
**Survey location**	
In person	102 (8.7)
Online	1,069 (91.3)

**Table 2.** The mean (± SD) adoptability ratings by label and description type. Participants (n = 1,171) rated how likely they were to adopt an animal from 0 (no thanks) to 100 (yes please)

Variable	Adoptability rating
**Label**	
Label present	54.06 (± 33.00)
No label	55.95 (± 31.99)
**Description**	
Affectionate	62.45 (± 31.37)
Calm	63.82 (± 30.94)
Eager to Make You Proud	59.11 (± 33.02)
Easy-Going	59.69 (± 32.73)
Energetic	48.64 (± 33.11)
Friendly	59.95 (± 33.07)
Lively	59.13 (± 31.56)
Non-Dominant	63.63 (± 31.47)
Sociable	62.43 (± 30.15)
No Description	51.73 (± 32.31)

### Effect of breed labels and behaviour descriptors

**Figure 2.**
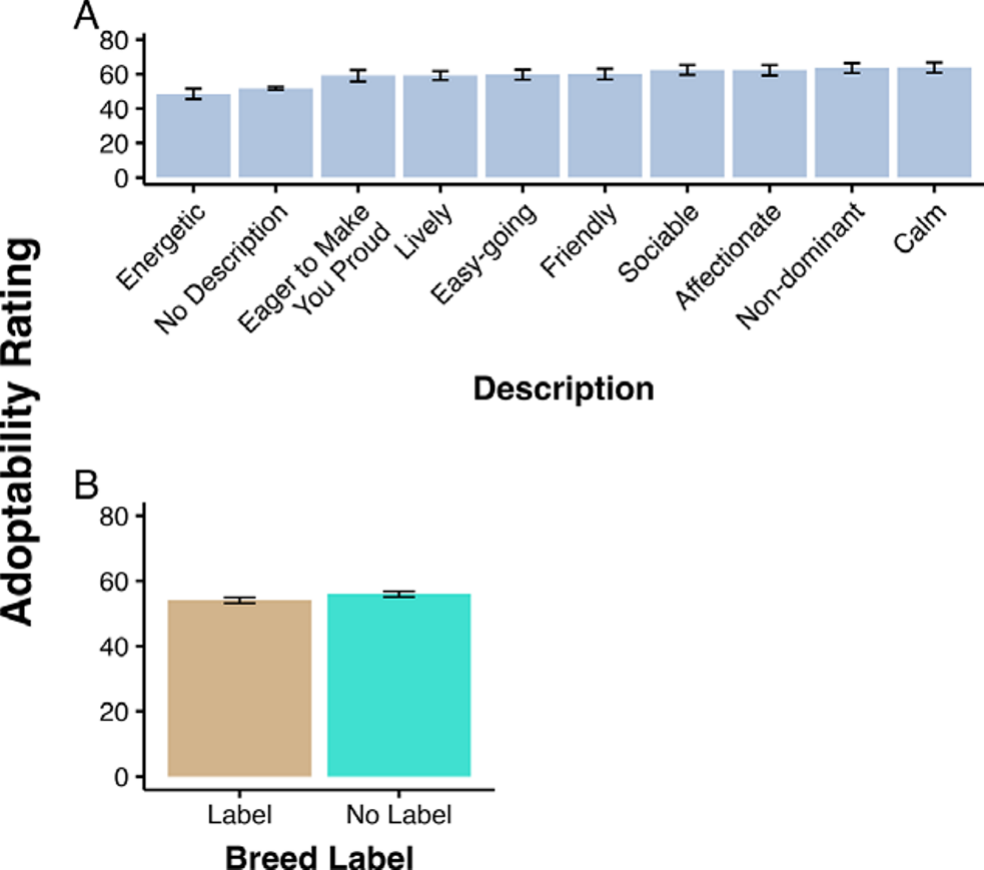
Average adoptability ratings by (A) description and (B) breed label. Possible adoptability ratings ranged from 0 to 100. Bars show the mean and error bars show the 95% confidence interval of the mean. Adoptability ratings (A) were significantly predicted by descriptions (*F*_9,8573_ = 43.08; *P* < 0.001) and breed label (B) (*F*_1,8604_ = 9.58; *P* = 0.002). All descriptions, except *energetic*, improved adoptability ratings compared to no description (all *P* < 0.001). A description of *energetic* led to lower adoptability ratings compared to all other descriptions (all *P* < 0.001). Overall, having a breed label predicted significantly lower adoptability ratings (*F*_1,8604_ = 9.58; *P* = 0.002).

The linear mixed effect model predicting adoptability from the presence/absence of a breed label, the type of description, and the location of the survey indicated that adoptability was significantly related to breed label (Hypothesis 1; *F*_1,8604_ = 9.58; *P* = 0.002) and description (Hypothesis 3; *F*_9,8573_ = 43.08; *P* < 0.001) presence. However, no interaction was found between breed label and description (*F*_9,8594_ = 0.66; *P* = 0.75), nor an effect of survey location (online or at the shelter; *F*_1,1158_ = 0.03; *P* = 0.86). Overall, the model had adequate explanatory power (conditional *R*^2^ = 0.51). However, the explanatory power from the fixed effects (*R*^2^ = 0.02) was low. The majority of the variance (44%) was explained by the random effect of individual participant. Picture ID accounted for 6% of the variance. Figure 2 illustrates the statistically significant effects on adoptability: the behavioural descriptions provided and the presence of a breed label.

The overall effect of adding a breed label led to lower adoptability ratings compared to not having a breed label (Figure 2). To further explore the effect of descriptions, Tukey-adjusted *post hoc* tests from the mixed effect model were conducted to compare the description types. All descriptions improved adoptability ratings compared to no description (all *P* < 0.001), with the exception of *energetic.* A description of *energetic* led to lower adoptability ratings compared to all other descriptors (all *P* < 0.001), and even lower than the model-estimated mean rating for having no description (*P* = 0.006).

To further explore the effect of the breed label, we conducted an independent regression for each breed label to evaluate whether specific breed labels influenced adoptability (Hypothesis 2).

**Figure 3.**
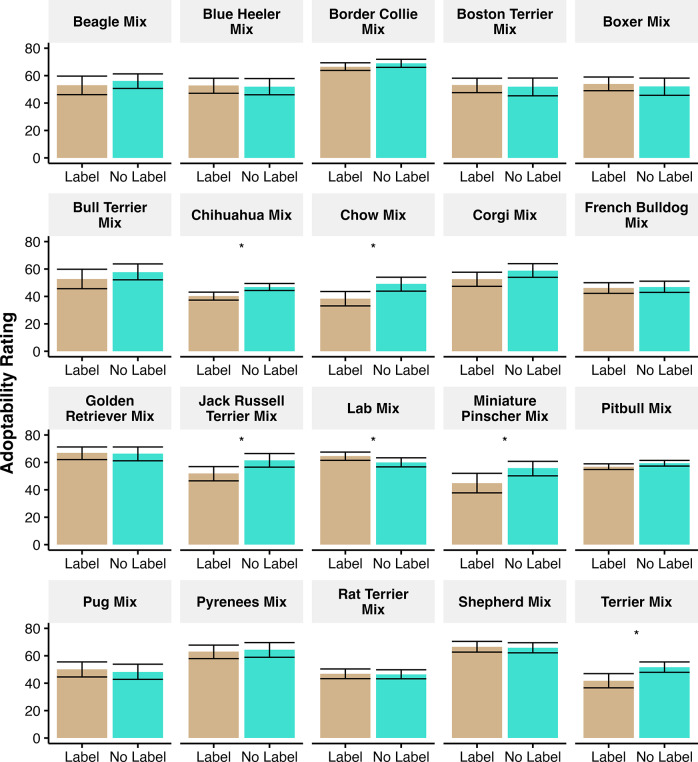
Average adoptability ratings for each breed with and without a label. Possible adoptability ratings ranged from 0 to 100. Bars show the mean and error bars show the bootstrap estimated 95% confidence interval of the mean. * Indicates a *P* < 0.05 on an independent regression. Having a breed label predicted lower adoption ratings for Chihuahua mixes, Chow mixes, Jack Russell Terrier mixes, Miniature Pinscher mixes, and Terrier mixes, and predicted higher adoptability ratings for Lab mixes.

Figure 3 shows the comparison between the presence and absence of a breed label for each individual breed name used. This analysis demonstrates that for most of the breed names used, the label had minimal impact, but having a label did lead to lower adoption ratings for Chihuahua mixes, Chow mixes, Jack Russell Terrier mixes, Miniature Pinscher mixes, and Terrier mixes. Having the breed label only helped (improved adoptability ratings) for Lab mixes and had no statistically significant impact on the remaining breeds.

**Figure 4.**
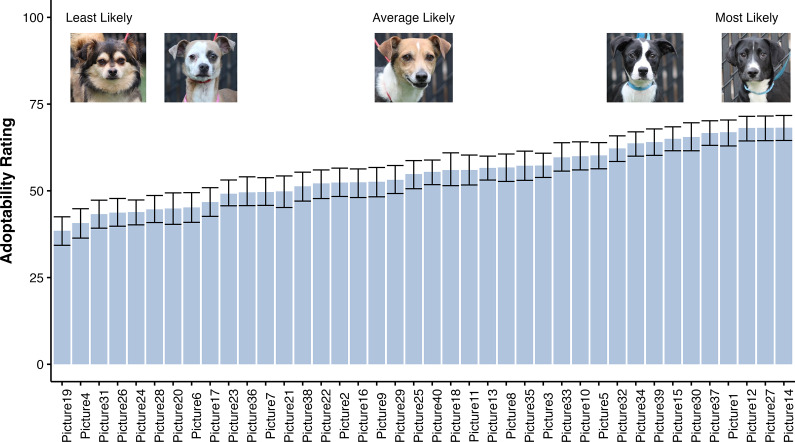
Average adoptability ratings for each picture and examples of least, average, and most likely to adopt dogs. Bars show the mean and error bars show the bootstrap estimated 95% confidence intervals of the mean. All the photos are included in the Supplementary material.

Lastly, Figure 4 illustrates the effect of the picture alone on overall adoptability ratings. Certain photos were rated as more adoptable than others (for the pictures of each of these dogs, see Supplementary material). Photos of the least, average, and most adoptable are provided in Figure 3. These results highlight that, in the absence of descriptors or labels, morphological characteristics account for important variability in adoptability ratings.

## Discussion

This study experimentally evaluated the combined and independent effects of breed labels and behavioural descriptors on perceived adoptability of shelter dogs. We hypothesised that: (1) dogs would be perceived as significantly more adoptable when shown without a breed label; (2) dogs with specific breed labels (e.g. pit bull mix) would be perceived as significantly less adoptable compared to other breed labels; and (3) having a description would make a dog seem significantly more adoptable.

The results from the present study indicate that photos of dogs without a breed label are perceived as more adoptable than those with a breed label, supporting Hypothesis 1. This adds to the growing body of evidence that removing breed labels may positively impact attractiveness and adoptability, with the exception of the label, ‘Lab mix’ (Gunter *et al.* 2016; Cohen *et al.* 2020; Reese 2021; Weseley-Jones 2022). Although the results were statistically different, it should be noted that the difference between the mean adoptability ratings for dogs with a breed label and those without was only 1.89 points (out of a possible 100). However, given the alternative to adoption is a longer length of stay or even euthanasia, it could be argued that anything that increases adoption interest, even slightly, should be considered.

Hypothesis 2 was partially supported as the presence of specific breed labels was correlated with lower adoptability ratings. Having a Chihuahua, Chow, Jack Russell Terrier, Miniature Pinscher, or Terrier mix label negatively impacted perceived adoptability. This contrasts with previous research at two private shelters in the US Pacific Northwest, which found that dogs in the Toy (e.g. Chihuahua and Miniature Pinscher) and Terrier (e.g. Jack Russell Terrier) groups had the shortest time until adoption, and dogs in the Bully group (e.g. Pit Bulls) had the longest length of stay (Svoboda & Hoffman 2015). Similarly, two separate studies in California and Florida found that dogs in the Lap group (e.g. Chihuahua and Miniature Pinscher) had the greatest adoption rate (Lepper *et al.* 2002; Protopopova *et al.* 2012).

In the current study, having a Pit Bull breed label did not decrease the adoptability rating, contrary to our prediction and much of the existing literature. One possible explanation is that participants may not have been making ratings under the same conditions or pressures as a real adoption decision. In an online or survey-based context, individuals might evaluate dogs more on perceived attractiveness in the moment rather than weighing long-term considerations such as breed restrictions, insurance limitations, or public perception, all of which can influence real-world adoption choices for Pit Bull-type dogs.

Having a breed label positively benefited one breed: Lab mixes. Labrador Retrievers are part of the American Kennel Club Sporting group (‘Sporting Group’ 2024). Research on the length of stay and adoptability of dogs labelled as Labrador Retrievers and dogs in the Sporting group has varied. In a study of 180 shelter dogs in Florida, dogs in the Sporting group had the longest median length of stay at 21.5 days compared to an overall median of 18 days (Protopopova *et al.* 2012). Although they took longer to place, a higher-than-average proportion of these dogs were ultimately adopted. Alternatively, in an exploration of over 70,000 dog adoption profiles in Australia, Nakamura *et al.* (2019) found that Labrador Retrievers had the shortest mean length of stay at 27.6 days compared to an overall average of 35.4 days. The inconsistencies in length of stay and adoption of dogs with certain breed labels across different studies could be yet another artifact of the inaccuracy of breed labelling using visual identification. In the present study, as well as in other studies examining the impact of breed on adoption, breed labels were assigned using visual identification (e.g. Protopopova *et al.* 2012; Svoboda & Hoffman 2015).

The results from the present study also partially supported Hypothesis 3. The presence of all descriptions, except *energetic*, had a positive impact on adoptability ratings. This aligns with previous findings from Nakamura *et al.* (2019), who indicated that specific personality traits, such as *lively* and *make you proud* were related to a decreased length of stay, whereas dogs with *energetic* in their personality descriptions had a longer length of stay. Notably, Nakamura *et al.* (2019) found significant interactions between certain personality traits and breeds. For instance, Australian cattle dogs labelled as *gentle* had longer lengths of stay, whereas Jack Russell terriers labelled as *gentle* had shorter lengths of stay. Additionally, although the average length of stay was longer for dogs with *energetic* in their description across breeds, it had a greater impact on some breeds compared to others. For example, having *energetic* in the description increased the average length of stay for Australian Cattle Dogs by 24.3 days, but only increased the average length of stay for Labrador Retrievers by 1.88 days. In contrast, the current study did not identify any significant interactions between breed labels and descriptions. This aligns with the findings of Weseley-Jones (2022), who also reported no interaction between breed label and descriptor. However, the current study extends those results by using multiple dog photos and a range of descriptions, as well as including conditions without breed labels or descriptions. These design differences may help explain why our study observed broader effects of descriptions overall, despite the lack of an interaction effect. The discrepancy in the interaction between breed label and description across studies may reflect methodological differences, as the present study and Weseley-Jones (2022) relied upon simulated adoption ratings rather than real-world outcomes. Regardless, the results suggest that certain descriptors can be used in adoption profiles to enhance perceived adoptability. Future research should explore the potential interaction between phenotypic characteristics and descriptions and their effect on perceived adoptability and actual adoption outcomes.

Previous research indicates that morphology is one of the most, if not *the* most important factor for why an adopter chooses a dog (Protopopova *et al.* 2012; Weiss *et al.* 2012; Protopopova & Wynne 2014, 2016). Our study further highlights the impact of morphology on adoptability. Interestingly, a dog with a long coat had the lowest adoptability rating, and a dog with short black hair had the highest adoptability rating. Early research on phenotypic predictors of adoption in shelter dogs suggested that black dogs were less preferred and less likely to be adopted (Wells & Hepper 1992; Posage *et al.* 1998). However, more recent research has suggested this may not be the case (Protopopova *et al.* 2012; Brown *et al.* 2013; Svoboda & Hoffman 2015). For instance, a study on photograph attributes in online adoption profiles found that black dogs with floppy ears had the shortest length of stay (Nakamura *et al.* 2020). Svoboda and Hoffman (2015) found that black dogs did not have significantly longer lengths of availability for adoption (LOA) nor higher rates of euthanasia compared to other coloured dogs, and Brown and colleagues (2013) found coat colour did not significantly impact length of stay at all.

The variation in morphological preferences observed across studies highlights significant individual variability in adopter preferences. This finding is further supported by the current study, where the largest proportion of variance in adoptability ratings was explained by the random effect of participant, suggesting that individuals differed in their overall willingness to adopt, regardless of specific breed labels or descriptions. This, combined with the wide range of adoptability ratings for individual dog photos, suggests that preferences for morphological traits may also vary across individuals. Morphology, as an immutable characteristic, cannot be altered, which makes this variability particularly encouraging. It suggests that for every morphological type, there may be an adopter whose preferences align. Thus, future research should focus upon how mutable elements of adoption profiles, such as body positioning (Isgate & Couchman 2018), photo background (Lamb *et al.* 2021), and the presence of other people, animals, or objects (Nakamura *et al.* 2020), can complement morphological traits to enhance adoptability.

A notable limitation of the present study is that adoption interest was measured as opposed to actual adoption outcomes. Participants were asked to rate the likelihood of adopting a dog based on images, breed labels, and descriptions, but these ratings do not necessarily translate into actual adoption outcomes. Factors such as in-person interactions (Protopopova & Wynne 2014) and shelter and municipality-specific policies (Hawes *et al.* undated; Weiss & Gramann 2009; Weiss *et al.* 2014) may also influence actual adoption outcomes, but were not accounted for in this study. Therefore, while our findings provide valuable insights into factors that might influence adoptability, they do not directly measure the actual likelihood of adoption.

While our study demonstrates that positive behavioural descriptors generally increase perceived adoptability, the exclusive use of positive traits represents an important limitation. In practice, descriptions that over-emphasise positive qualities may create unrealistic expectations for adopters. This mismatch could lead to disappointment and, in some cases, the dog being returned to the shelter, which can negatively affect animal welfare. A survey of recent dog adopters found that owners with higher expectations for their dog’s behaviour were significantly more likely to return their dog (Powell *et al.* 2022a). Additionally, owners who returned their pet due to unrealistic expectations were less likely to adopt another pet within the year (Powell *et al.* 2022b). Therefore, it is important that adoption profile descriptions should balance attractiveness with accuracy, ensuring that descriptors provide potential adopters with a realistic understanding of the dog’s personality and behaviour to increase the likelihood of long-term placements.

### *Animal welfare implication*s

This study identifies practical strategies that may help increase adoption interest and potentially increase adoption outcomes for shelter dogs. Removing breed labels, especially for stigmatised breeds, may reduce bias and increase adoption interest, potentially shortening length of stay and lowering euthanasia risk. Including positive behavioural descriptors, such as *calm* or *affectionate*, can further enhance adoptability by helping adopters connect with dogs’ personalities. Since physical traits cannot be altered, focusing on how dogs are presented through language and imagery offers a humane, low-cost way to promote welfare and increase adoption success.

## Conclusion

The results from the present study add to the growing body of research supporting the removal of breed labels and the inclusion of behavioural descriptions in shelter dog profiles. The study also indicates that there is a lot of individual variability in morphological preferences in shelter dogs. Future research should continue to explore the complex interactions between morphology, descriptions, and individual preferences to identify effective strategies for attracting potential adopters and increasing adoption rates.

## Supporting information

Archer et al. supplementary material Archer et al. supplementary material

## Supplementary material

The supplementary material for this article can be found at http://doi.org/10.1017/awf.2025.10043.
