# Supplementary material for: The role of breed and personality descriptions in influencing perceptions of shelter dog adoptability
Source: Anim Welf. 2025 Nov 17;34:e75. doi: 10.1017/awf.2025.10043 (PMC12645507; doi:10.1017/awf.2025.10043)
Supplement: Archer et al. supplementary material [file S0962728625100432sup001.pdf]

The role of breed and personality descriptions in influencing perceptions of shelter dog adoptability: Supplementary material

Courtney A Archerhttps://orcid.org/0000-0003-1601-854X[1], Nathaniel J Hall[2], and Allison Andrukonis[3]

[1] University of Minnesota, Animal Sciences, 1364 Eckles Ave, St Paul, MN, 55108, USA

[2] Texas Tech University, Animal and Food Sciences, Indiana Avenue, Lubbock TX 79409, USA

[3] University of Wisconsin-Madison, Department of Animal and Dairy Sciences, 1675 Observatory Dr, Madison, WI, 53706 USA

Author for correspondence: Allison Andrukonis, email: andrukonis@wisc.edu

Pictures with associated breed labels and mean (± SD) adoptability rating (regardless of the presence of a breed label)

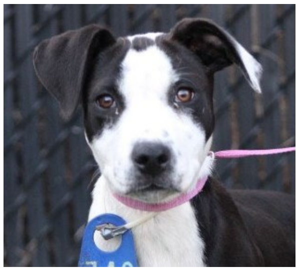

Picture 1
Border Collie Mix
66.92 (± 29.19)

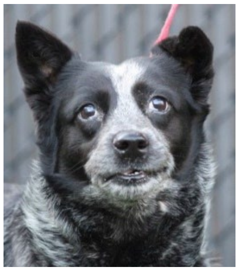

Picture 2
Blue Heeler Mix
53.40 (± 31.54)

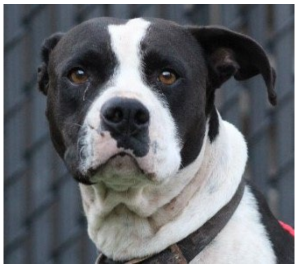

Picture 3
Pitbull Mix
57.28 (± 32.12)

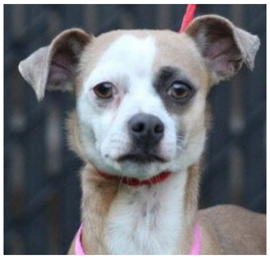

Picture 4
Chihuahua Mix
40.72 (± 33.09)

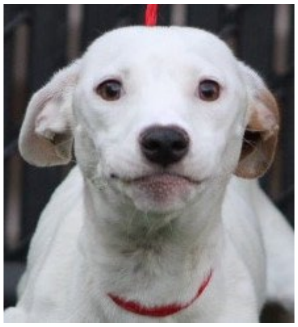

Picture 5
Lab Mix
60.22 (± 30.50)

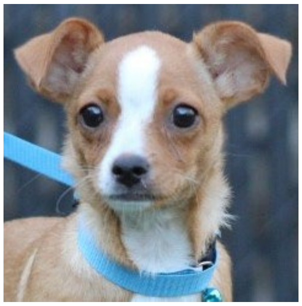

Picture 6
Chihuahua Mix
45.24 (± 35.08)

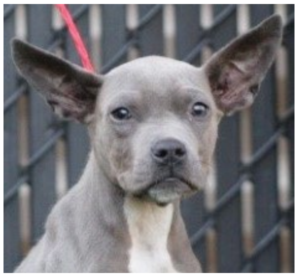

Picture 7
French Bulldog Mix
49.65 (± 31.76)

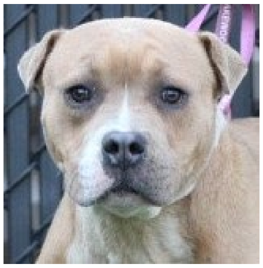

Picture 8
Pitbull Mix
56.73 (± 33.26)

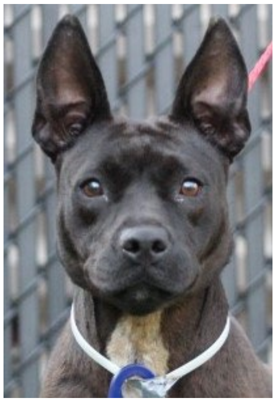

Picture 9
Boston Terrier Mix
52.60 (± 31.66)

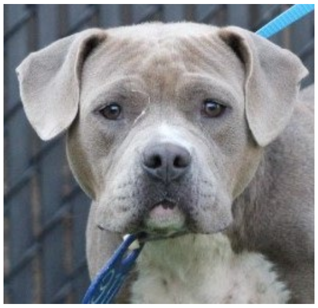

Picture 10
Pitbull Mix
59.98 (± 34.68)

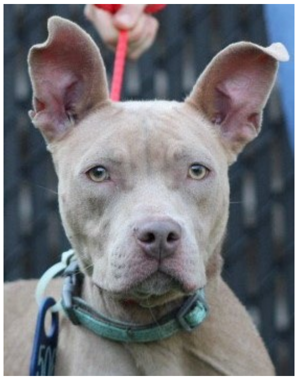

Picture 11
Pitbull Mix
56.02 (± 34.73)

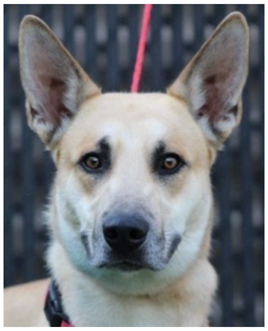

Picture 12
Shepherd Mix
68.13 (± 29.11)

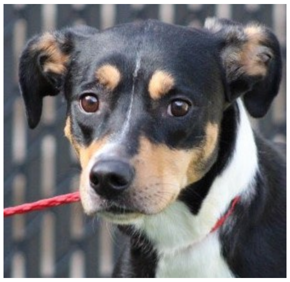

Picture 13
Jack Russell Terrier Mix
56.63 (± 29.43)

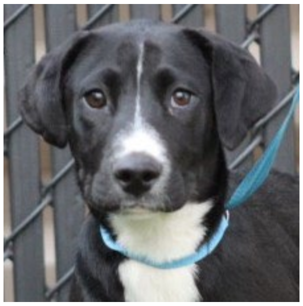

Picture 14
Border Collie Mix
68.20 (± 28.74)

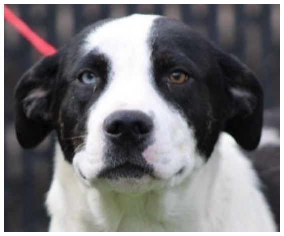

Picture 15
Pitbull Mix
65.00 (± 28.40)

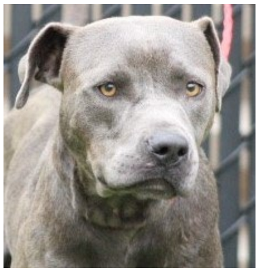

Picture 16
Pitbull Mix
52.45 (± 33.11)

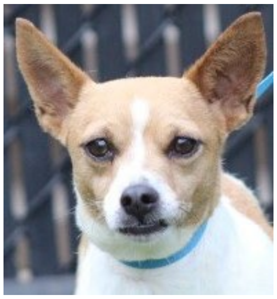

Picture 17
Terrier Mix
46.75 (± 31.25)

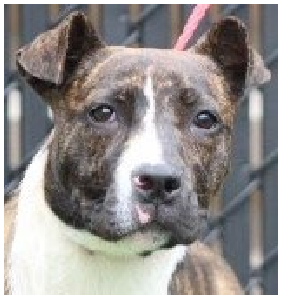

Picture 18
Bull Terrier Mix
55.97 (± 32.31)

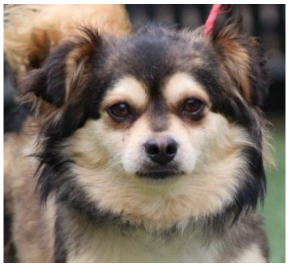

Picture 19
Chihuahua Mix
38.40 (± 31.90)

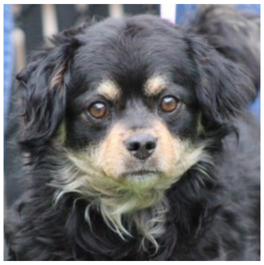

Picture 20
Chihuahua Mix
44.89 (± 33.49)

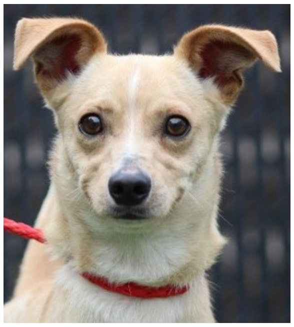

Picture 21
Terrier Mix
49.83 (± 33.64)

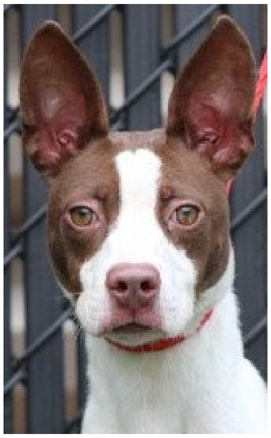

Picture 22
Miniature Pinscher Mix
52.13 (± 31.46)

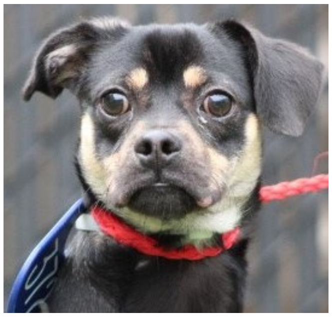

Picture 23
Pug Mix
49.19 (± 32.88)

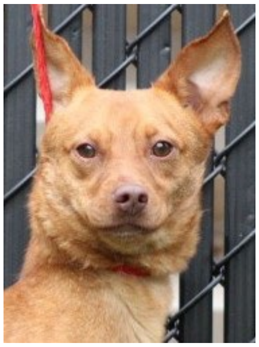

Picture 24
Chow Mix
43.88 (± 30.60)

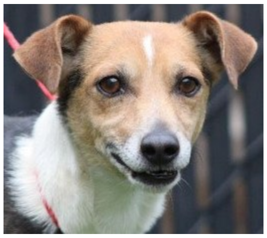

Picture 25
Beagle Mix
54.83 (± 30.37)

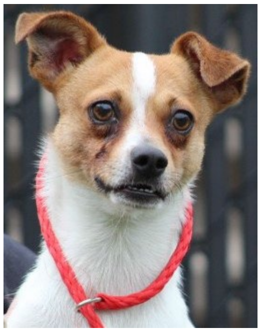

Picture 26
Rat Terrier Mix
43.71 (± 31.85)

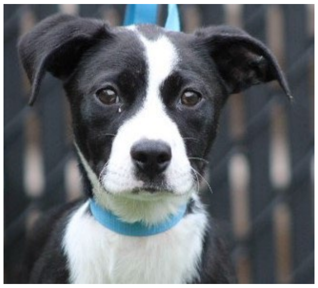

Picture 27
Border Collie Mix
68.16 (± 28.02)

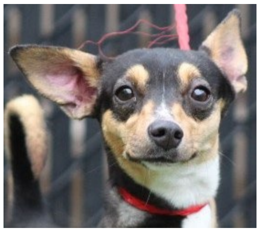

Picture 28
Rat Terrier Mix
44.68 (± 34.42)

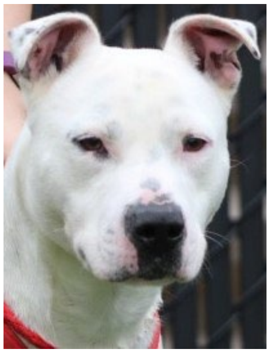

Picture 29
Boxer Mix
53.16 (± 32.53)

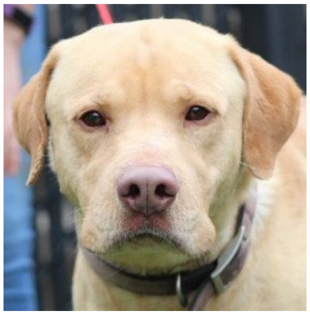

Picture 30
Lab Mix
65.51 (± 28.05)

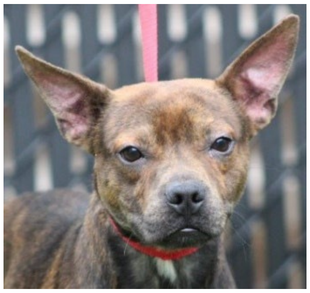

Picture 31
French Bulldog Mix
43.27 (± 30.79)

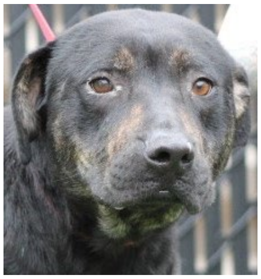

Picture 32
Lab Mix
62.24 (± 31.25)

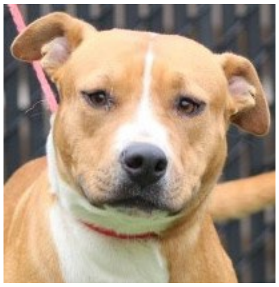

Picture 33
Pitbull Mix
59.68 (± 32.33)

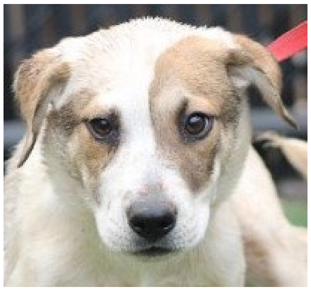

Picture 34
Pyrenees Mix
63.70 (± 28.19)

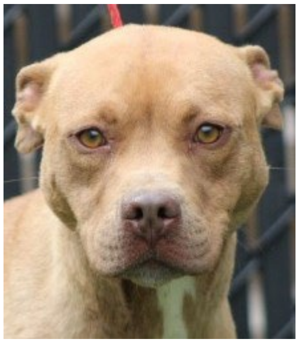

Picture 35
Pitbull Mix
57.25 (± 33.44)

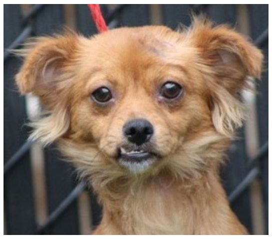

Picture 36
Chihuahua Mix
49.59 (± 33.90)

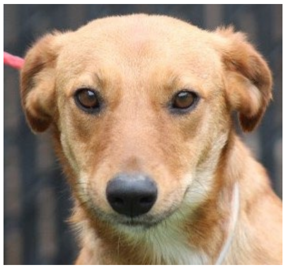

Picture 37
Golden Retriever Mix
66.67 (± 27.94)

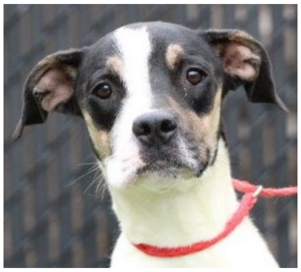

Picture 38
Rat Terrier Mix
51.30 (± 32.28)

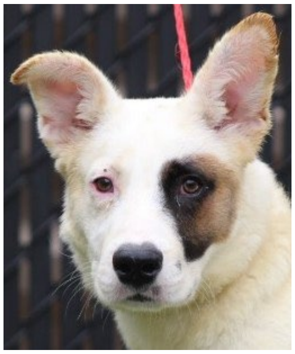

Picture 39
Shepherd Mix
64.04 (± 30.04)

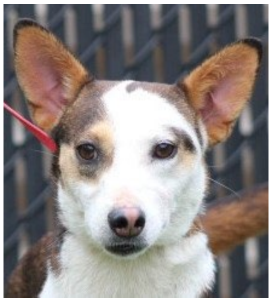

Picture 40
Corgi Mix
55.48 (± 33.09)
